# Ceftriaxone-Resistant *Salmonella enterica* Serotype Newport, France

**DOI:** 10.3201/eid1406.071168

**Published:** 2008-06

**Authors:** Svetlana Egorova, Mohammed Timinouni, Marie Demartin, Sophie A. Granier, Jean M. Whichard, Vartul Sangal, Laëtitia Fabre, Aurélia Delauné, Maria Pardos, Yves Millemann, Emmanuelle Espié, Mark Achtman, Patrick A.D. Grimont, François-Xavier Weill

**Affiliations:** * Institut Pasteur, Paris, France; † Agence Française de Sécurité Sanitaire des Aliments, Maisons-Alfort, France; ‡ Centers for Disease Control and Prevention, Atlanta, Georgia, USA; § Max-Planck-Institute für Infektionsbiologie, Berlin, Germany; ¶ Ecole Nationale Vétérinaire d’Alfort, Maisons-Alfort, France; # Institut de Veille Sanitaire, Saint-Maurice, France; ** University College, Cork, Ireland; 1 Current affiliation: Institut Pasteur, St. Petersburg, Russia.; 2 Current affiliation: Institut Pasteur, Casablanca, Morocco.

**Keywords:** Salmonella enterica, multidrug resistance, ceftriaxone, serotype Newport, CMY-2, France, dispatch

## Abstract

The multidrug-resistant (MDR) *Salmonella enterica* serotype Newport strain that produces CMY-2 β-lactamase (Newport MDR-AmpC) was the source of sporadic cases and outbreaks in humans in France during 2000–2005. Because this strain was not detected in food animals, it was most likely introduced into France through imported food products.

Third-generation cephalosporins are drugs of choice for treatment of persons with nontyphoidal *Salmonella* infections that require chemotherapy or when fluoroquinolones are contraindicated. A new public health concern is the emergence of third-generation cephalosporin–resistant *Salmonella* isolates (*1*). Multidrug-resistant (MDR) *Salmonella enterica* serotype Newport isolates that produce CMY-2, a β-lactamase that inactivates third-generation cephalosporins, were first reported in the United States in 1998 (*2*). These isolates, known as Newport MDR-AmpC, have quickly spread through the United States in cattle and humans (*3*–*5*). It has been hypothesized that use of ceftiofur, a third-generation cephalosporin licensed in the United States for use in cattle, could have selected for Newport MDR-AmpC (*2*–*4*,*7*). Several observations and case-control studies suggested beef and milk from dairy cattle were substantial sources of Newport MDR-AmpC infection in humans (*6*–*8*).

These isolates seem to be extremely rare in Europe. Two surveys performed in England and Wales (278,308 human *Salmonella* isolates tested, 1992–2003) and Spain (959 human *Salmonella* isolates, 1999–2000) did not detect Newport MDR-AmpC (*9*,*10*). In St. Petersburg, Russia, only 1 Newport MDR-AmpC isolate was reported among 1,078 *Salmonella* isolates during 2002–2005 (*11*). In France, a small outbreak (14 cases) of Newport MDR-AmpC was detected in 2003 and linked to consumption of imported horse meat (*12*). We undertook the present study to acquire more knowledge on circulation of Newport MDR-AmpC in humans, animals, and animal-derived food in France.

## The Study

From 2000 through 2005, the French National Reference Centre for *Salmonella* at the Institut Pasteur in Paris reported 829 Newport isolates among 69,759 *Salmonella* clinical isolates. During this period and depending on the year, serotype Newport ranked between 6th and 10th in prevalence among human serotyped isolates. From 2000 through 2005, the Agence Française de Sécurité Sanitaire des Aliments reported 2,160 Newport isolates among 101,791 *Salmonella* isolates collected from animals and food products.

Antimicrobial drug susceptibility testing was performed on 585 human Newport isolates and 342 nonhuman Newport isolates by disk diffusion with 32 antimicrobial drugs (additional information available from fxweill@pasteur.fr). Data for Newport human isolates are shown in the Table. Of 585 isolates tested, 46 (7.9%) were resistant to third-generation cepalosporins. The geographic origin of the isolates was mainly the Paris metropolitan area and northern France (Appendix Table). There was a high prevalence of third-generation cephalosporin–resistant isolates during 2000 (15%) and 2003 (17.5% caused by a small outbreak). No third-generation cephalosporin resistance was detected in any of the nonhuman Newport isolates tested.

**Table:** Resistance to specific antimicrobial drugs in *Salmonella enterica* serotype Newport from humans in France, 2000–2005*

Drug	% Resistant isolates					
	2000 (n = 100) (N = 109)	2001 (n = 124) (N = 134)	2002 (n = 66) (N = 71)	2003 (n = 126) (N = 138)	2004 (n = 91) (N = 94)	2005 (n = 78) (N = 80)
Amoxicillin	27	9.7	1.5	19.8	8.8	3.8
Ceftriaxone/ceftazidime	15	4	1.5	17.5	2.2	0
Gentamicin	4	1.6	0	1.6	2.2	0
Nalidixic acid	23	7.3	4.5	1.6	4.4	2.6
Ciprofloxacin	0	0	0	0	0	0
Sulfonamides	29	10.5	4.5	19.8	8.8	0
Trimethoprim	10	4	3	1.6	4.4	0
Chloramphenicol	25	9.7	1.5	15.9	8.8	0
Tetracycline	27	11.3	3	19	9.9	3.8

*n, no. of isolates studied; N, no. of isolates received at the French National Reference Centre for *Salmonella* (1 per patient).

Experiments were performed on the 46 third-generation cephalosporin–resistant Newport isolates (additional information available from fxweill@pasteur.fr). All but 1 of the Newport isolates were resistant to cefoxitin (Appendix Table). These isolates showed 4 resistance phenotypes; most (41, 89.1%) were resistant to streptomycin, sulfonamides, chloramphenicol, and tetracycline. PCR and sequencing showed that the 45 isolates resistant to cefoxitin were positive for the *bla*_CMY-2_ gene, and cefoxitin-susceptible isolates contained the extended-spectrum β-lactamase gene *bla*_CTX-M-1_. Ceftriaxone MICs of Newport MDR-AmpC isolates ranged from 32 mg/L to >256 mg/L, and ceftazidime MICs ranged from 64 mg/L to >256 mg/L. No *bla*_TEM_ genes were detected. Three isolates with additional resistance to aminoglycosides contained a class 1 integron with the 1-kb gene cassette *aadA24* (known to encode resistance to streptomycin and spectinomycin) (*11*). The chloramphenicol/florfenicol resistance gene *floR* was detected in all but 1 CMY-2–producing Newport isolate.

**Figure 1:**
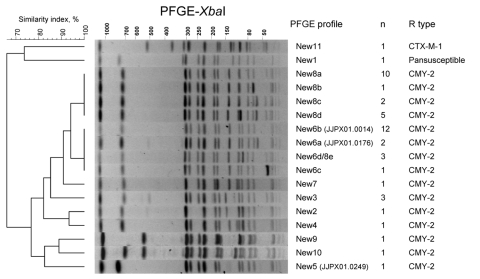
Representative *Xba*I pulsed-field gel electrophoresis (PFGE) profiles of third-generation cephalosporin–resistant *Salmonella* Newport isolates studied. A dendrogram was generated with Bionumerics software (Applied Maths, Sint-Martens-Latem, Belgium). The PFGE profile (and if there were indistinguishable isolates in the PulseNet USA database [www.cdc.gov/pulsenet], the corresponding Centers for Disease Control and Prevention PulseNet profile), the number of isolates, and the β-lactamase genes are indicated.

**Figure 2:**
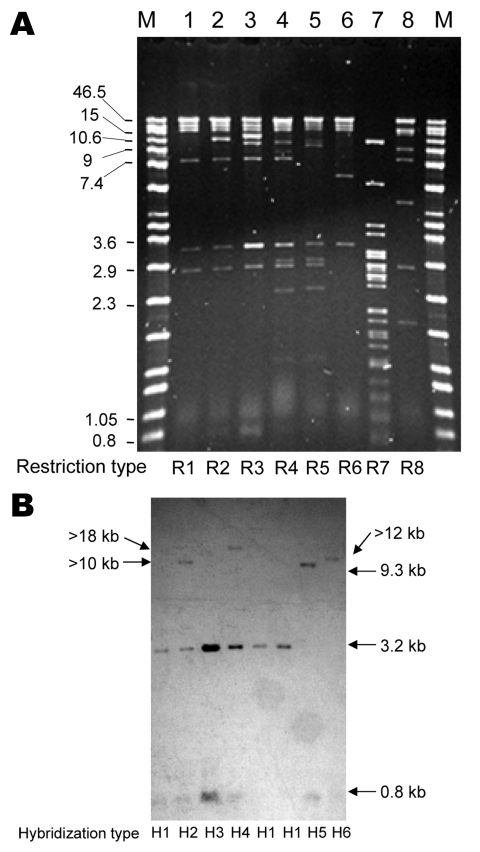
Representative *Pst*I restriction profiles (A) and *bla*_CMY-2_ Southern hybridization (B) of plasmids from *Escherichia coli* DH10B transformants of CMY-2–producing *Salmonella* spp. clinical isolates. Lane M, Raoul molecular mass marker (Qbiogene, Illkirch, France). Lane 1, DH10B/00-7490; lane 2, DH10B/03-3349; lane 3, DH10B/03-3367; lane 4, DH10B/00-3525; lane 5, DH10B/00-4165; lane 6, DH10B/03-9969; lane 7, DH10B/03-9243; lane 8, DH10B/02-2049. Values on the left of panel A are in kb. Restriction and hybridization profiles are indicated. The gel is focused on the resolution of high molecular mass bands; smaller bands (in particular, the 0.8-kb band) are not well visualized.

Clonal relatedness of Newport isolates was assessed by multilocus sequence typing (MLST) and PulseNet standard method pulsed-field gel electrophoresis (PFGE) (Figure 1). All 16 Newport MDR-AmpC isolates tested had a common sequence type (ST), ST45. *Xba*I-PFGE identified 10 distinct profiles (similarity 76.7%) among all 45 Newport MDR-AmpC isolates. Single enzyme matches were found for 3 of the profiles (15 isolates) in the US PulseNet national database (www.cdc.gov/pulsenet; Appendix Table; Figure 2). Two PFGE types (New6 and New8) were divided into 2–4 subtypes because of additional band(s) <100 kb. Isolates from the 2003 outbreak showed 4 similar but distinct PFGE profiles that differed by 1–2 bands, migrated between 60 and 100 kb, and were attributed to plasmid(s) (additional information available from fxweill@pasteur.fr). If only cases with indistinguishable PFGE profiles had been tested, potentially related cases would not have been linked to this outbreak. Therefore, during an outbreak investigation of Newport MDR-AmpC, analysis of plasmid content (either by alkaline lysis or S1 nuclease, depending on size of additional bands) might complete *Xba*I-PFGE profiles for isolates whose profiles differ by 1 or 2 additional bands of low molecular mass.

Alkaline lysis extraction showed that all but 1 of the Newport MDR-AmpC isolates harbored a plasmid >125 kb that hybridized with a *bla*_CMY-2_ probe; the remaining isolate harbored a plasmid of 100 kb (Appendix Table). Analysis with S1 nuclease showed that these plasmids were 100 kb–370 kb. Up to 3 additional plasmids (3.5 kb–100 kb) that did not have *bla*_CMY-2_ were detected in most isolates (Appendix Table). Cephalosporin resistance was transferred by electroporation of plasmid DNA to *Eschericha coli* DH10B for all 38 CMY-2–positive isolates tested. When present in the donor strain, resistance to sulfonamides, chloramphenicol, and tetracycline was also transferred. Restriction analysis of plasmids isolated from transformants showed 6 similar restriction profiles for Newport isolates (R1–R6) (Figure 2, Appendix Table). R1 was predominant (found in 26 isolates among 35 tested, 74.3%). Newport plasmids R1–R6 and Agona plasmid R8 were shown by PCR to contain variant A/C_2_ replicons (*13*), whereas Typhimurium plasmid R7 contained the I1 replicon.

*Pst*I-digested plasmids analyzed by Southern hybridization with a *bla*_CMY-2_ probe (Figure 2) showed 4 hybridization profiles among Newport isolates. Profile H1 corresponded to plasmid type C described by Carattoli et al. (*14*). Profiles H2, H3, and H4 differed from H1 by 1 additional band (>10 kb for H2, 3.2 kb for H3, and >18 kb for H4), which indicated that the *bla*_CMY-2_ gene was partially or totally duplicated.

## Conclusions

Newport MDR-AmpC isolates have been the source of sporadic cases and small outbreaks in humans in France during 2000–2005. All isolates had the same MLST type, ST45, and highly similar *Xba*I-PFGE profiles. Their plasmids carrying *bla*_CMY-2_ were homogeneous (same incompatibility group A/C_2_, a main restriction type R1, and a main hybridization type H1). These results support clonal expansion of 1 Newport strain (or a limited number of genetically related Newport strains) able to acquire and maintain a large incA/C_2_ MDR plasmid.

The source of the French isolates remains unknown. However, this strain was not found in French food animals or domestically produced food products (additional information available from fxweill@pasteur.fr). One outbreak during the study period was linked to imported horse meat. Further investigation identified the source as a wholesaler who imported meat from Belgium, the United Kingdom, Hungary, Canada, Brazil, Argentina, Uruguay, and Australia (*12*). In contrast to Europe, Newport MDR-AmpC has been frequently seen in the United States during the past decade. Furthermore, several characteristics were shared between US and French Newport MDR-AmpC isolates: ST45 (*15*), PFGE profiles New5, New6a, and New6b (displayed by 15 isolates among the 45 studied), and *bla*_CMY-2_ plasmid hybridization type H1 (*14*). We can reasonably hypothesize that during 2000–2005 some isolates likely entered France from North America through imported food. Alternatively, they could have come to France and North America from some other country.

## Supplementary Material

Appendix Table Characteristics of Samonella spp. isolates used in this study*
