# Supplementary material for: Ceftriaxone-Resistant Salmonella enterica Serotype Newport, France
Source: Emerg Infect Dis. 2008 Jun;14(6):954–7. doi: 10.3201/eid1406.071168 (PMC2600296; doi:10.3201/eid1406.071168)
Supplement: Appendix Table — Characteristics of Samonella spp. isolates used in this study* [file 07-1168_appT-s1.pdf]

Appendix Table. Characteristics of *Samonella* spp. isolates used in this study*

Isolate	Date of isolation	Area of isolation†	Patient age group‡, sex	Source	Antimicrobial drug resistance phenotype§	Class 1 integron size, kb (gene cassette)	PFGE profile¶	MLST type	Plasmid profile#	Plasmid restriction profile**
S. Newport isolates										
00-3525	2000 Jun	75	III-F	Stool	AFoxCazSSuCTe	_	New6b (JJPX01.0014)	ST45	100, 50, 3.5	R4
00-3767	2000 Jun	60	III-F	Stool	AFoxCazSSuCTe	_	New2	ST45	>125, 3.5	R3
00-3784	2000 Jun	75	II-M	Stool	AFoxCazSSpKToGSuCTe	1 (*aadA24*)	New3	ST45	>125, 7	R1
00-4165	2000 Jul	62	II-M	Stool	AFoxCazSSpKToGSuCTe	1 (*aadA24*)	New 3	ST45	>125, 7	R5
00-4652	2000 Jul	60	II-F	Stool	AFoxCazSSuCTe	_	New 4	ND	>125, 3.5	R4
00-5089	2000 Jul	2A	II-F	Stool	AFoxCazSSpKToGSuCTe	1 (*aadA24*)	New 3	ST45	>125, 7	R1
00-6399	2000 Sep	92	V-M	NK	AFoxCazSSuCTe	_	New5 (JJPX01.0249)	ND	>125, 3.5	R1
00-7093	2000 Sep	77	II-F	Stool	AFoxCazSSuCTe	_	New 7	ST45	>125, 90	R1
00-7098	2000 Sep	93	II-F	Stool	AFoxCazSSuCTe	_	New6b (JJPX01.0014)	ND	>125, 90	R1
00-7298	2000 Sep	77	IV-F	Stool	AFoxCazSSuCTe	_	New6b (JJPX01.0014)	ND	>125, 90	R1
00-7325	2000 Sep	75	IV-F	Stool	AFoxCazSSuCTe	_	New6b (JJPX01.0014)	ST45	>125, 90	R1
00-7400	2000 Sep	77	IV-M	Stool	AFoxCazSSuCTe	_	New6b (JJPX01.0014)	ND	>125	R1
00-7490	2000 Sep	89	II-M	Stool	AFoxCazSSuCTe	_	New6b (JJPX01.0014)	ND	>125, 90	R1
00-7777	2000 Oct	77	II-F	NK	AFoxCazSSuCTe	_	New6b (JJPX01.0014)	ND	>125, 90	R1
00-8066	2000 Oct	93	II-F	Stool	AFoxCazSSuCTe	_	New6b (JJPX01.0014)	ND	>125, 90	R1
01-2010	2001 Apr	92	V-F	Stool	AFoxCazSSuCTe	_	New6a (JJPX01.0176)	ND	>125, 60, 3.5	R2
01-2288	2001 Arp	93	IV-F	Blood	AFoxCazSSuCTe	_	New6a (JJPX01.0176)	ST45	>125, 60, 3.5	ND
01-9637	2001 Dec	95	NK-F	Stool	AFoxCazSSuCTe	_	New6b (JJPX01.0014)	ND	>125, 3.5	R1
01-9867	2001 Dec	93	III-F	Stool	AFoxCazSSuCTe	_	New6b (JJPX01.0014)	ND	>125, 3.5	ND
01-10075	2001 Dec	77	II-M	Stool	AFoxCazSSuCTe	_	New6b (JJPX01.0014)	ND	>125, 3.5	ND
02-7891	2002 Oct	78	III-M	Stool	AFoxCazSSuCTe	_	New8a	ST45	>125, 3.5	R1
03-3125	2003 May	62	II-F	Stool	AFoxCazSSuCTe	_	New8b	ND	>125, 70, 3.5	R2
03-3136	2003 May	62	III-F	Stool	AFoxCazSSuCTe	_	New8a	ND	>125, 3.5	R1
03-3179	2003 May	62	III-M	Stool	AFoxCazSSuCTe	_	New8c	ND	>125, 60	R1
03-3184	2003 May	62	IV-F	Stool	AFoxCazSSuCTe	_	New8a	ST45	>125, 3.5	R2
03-3222	2003 May	62	V-F	Blood	AFoxCazSSuCTe	_	New8a	ND	>125, 3.5	ND
03-3224	2003 May	59	IV-M	Stool	AFoxCazSSuCTe	_	New8a	ND	>125, 3.5	ND
03-3225	2003 May	59	IV-F	Stool	AFoxCazSSuCTe	_	New8a	ND	>125, 3.5	R1
03-3243	2003 May	91	III-M	Stool	AFoxCazSSuCTe	_	New8a	ND	>125, 3.5	ND
03-3265	2003 May	62	II-M	Stool	AFoxCazSSuCTe	_	New8d	ND	>125, 100, 60, 3.5	ND
03-3349	2003 May	62	NK-F	Stool	AFoxCazSSuCTe	_	New8d	ST45	>125, 100, 60, 3.5	R2
03-3350	2003 May	62	II-M	Stool	AFoxCazSSuCTe	_	New8c	ND	>125, 60, 3.5	R1
03-3465	2003 May	59	V-M	Stool	AFoxCazSSuCTe	_	New8a	ND	>125, 3.5	ND
03-3519	2003 May	59	II-F	Stool	AFoxCazSSuCTe	_	New8d	ND	>125, 100, 60, 3.5	R1
03-3603	2003 Jun	59	III-M	Stool	AFoxCazSSuCTe	_	New8d	ND	>125, 100, 60, 3.5	R1
03-3642	2003 Jun	92	IV-NK	Stool	AFoxCazSSuCTe	_	New8a	ND	>125, 3.5	ND
03-4620	2003 Jul	62	IV-M	Stool	AFoxCazSSuCTe	_	New8a	ND	>125, 3.5	R1
03-5145	2003 Jul	59	III-M	Stool	AFoxCazSSuCTe	_	New8d	ST45	>125, 100, 60, 3.5	R1
03-6521	2003 Sep	59	IV-M	Stool	AFoxCazSSuCTe	_	New6d/8e	ST45	>125, 100, 3.5	R2
03-6773	2003 Sep	62	IV-F	Stool	AFoxCazSSuCTe	_	New6d/8e	ND	>125, 100, 3.5	R1
03-7268	2003 Sep	62	IV-M	Stool	AFoxCazSSuCTe	_	New6b (JJPX01.0014)	ND	>125, 100, 3.5	R1

03-7338	2003 Sep	62	IV-M	Stool	AFoxCazSSuCTe	_	New6d/8e	ST45	>125, 100, 3.5	R1
03-8748	2003 Nov	11	V-M	Stool	ACroSuTmp	ND	New9	ST118	ND	ND
03-9969	2003 Dec	59	V-F	Stool	AFoxCazSSuTe	_	New6c	ST45	>125, 40, 5	R6
04-4556	2004 Jul	11	V-F	Stool	AFoxCazSSuCTe	_	New9	ND	>125, 3.5	R1
04-9597	2004 Dec	95	V-F	Urine	AFoxCazSSuCTe	_	New10	ST45	>125, 3.5	R1
S. Newport reference strain										
50 K					Pan susceptible	ND	New1	ST31	ND	ND
CMY-2–producing *S*. Typhimurium isolate										
03-9243	2003 Oct	44	V-F	Stool	AFoxCazSKToGSuTmpCNal	–	STM53	ND	220, 90	R7
CMY-2–producing *S*. Agona isolates										
02-2049	2002 Apr	93	V-NK	Stool	AFoxCazSKSuTmpCTe	1.2 (*dfrA1-orfX*)	Ago1 (JABX01.0055)	ND	140	R8
02-2059	2002 Apr	77	V-F	Stool	AFoxCazSKSuTmpCTe	1.2 (*dfrA1-orfX*)	Ago1 (JABX01.0055)	ND	140	R8

*PFGE, pulsed-field gel electrophoresis; MLST, multilocus sequence typing; ST, sequence type; ND, not determined; NK, not known.

†Numbers are those of Départements (French administrative subdivisions) in the Paris metropolitan area (Départements 60, 75, 77, 78, and 91–95) and northern France (Départements 59 and 62).

‡I, <1 y; II, 1–5 y; III, 6–14 y; IV, 15–64-y; V, ≥65 y.

§A, amoxicillin; Fox, cefoxitin; Caz, ceftazidime; S, streptomycin; Su, sulfonamides; C, chloramphenicol; Te, tetracycline; K, kanamycin; To, tobramycin; G, gentamicin; Sp, spectinomycin; Cro, ceftriaxone; Tmp, trimethoprim; Nal, nalidixic acid.

¶Profiles in parentheses were obtained from the PulseNet USA database (www.cdc.gov/pulsenet).

#Determined by the alkaline lysis method.

**Profiles are shown in Figure 2.
